# Fertility intention among married women in Ethiopia: a multilevel analysis of Ethiopian demographic health survey 2016

**DOI:** 10.1186/s40834-022-00201-z

**Published:** 2023-01-18

**Authors:** Berhan Tsegaye Negash

**Affiliations:** Department of Midwifery, Collage of Health Science and Medicine, Hawassa University, Hawassa, Ethiopia

## Abstract

**Background:**

Fertility intention is the central aspect of countries which determine their population demography. Therefore, proportion and factors associated with fertility intention should be studied at different level of community for designing of appropriate policies, strategies, and programs. Despite its importance, information is scarce about proportion and predictors of fertility intention among women of reproductive age in Ethiopia, in 2016.

**Methods:**

A secondary data analysis was done on 2016 Ethiopian Demographic and Health Survey/EDHS/ in this study. A total of 1423 fecund, married, and sexually active women were included this study. Multilevel mixed-effect logistic regression model was done to show association between fertility desire and explanatory variables. Adjusted Odds Ratio with 95% Confidence Interval (CI) was computed to assess the strength and significance of association.

**Results:**

Prevalence of fertility intention was 63.5% (95%CI:62.2%,64.8%) in Ethiopia, in 2016. The response rate of this study was 100%. The odd of fertility desire was higher among women of age 20-34 years (AOR=2.5,95%CI:1.5,4.0), women of age 35-49 years (AOR= 9,95%CI:12.2,45.4), Muslim followers (AOR=5.4,95%CI:3.6,7.9), other religions followers (AOR= 1.8,95%CI:1.2,3.0), women who did not want to use modern contraceptive (AOR=3.1,95%CI:2.2,4.3). However, the likelihood of fertility intention was low among women who owned mobile phone (AOR=0.6,95%CI:0.4,0.87), and women with more than one partner (AOR=0.5,95%CI:0.41,0.8). At the community level factors like: Community education status (AOR= 1.67,95%CI:1.26,2.2) and region were factors strongly linked to fertility intention.

**Conclusions:**

In this study, prevalence of fertility desire was higher compared to other countries. Participants age, religion, intention to use modern contraceptive, own mobile, and having multiple partners were individual factors associated with fertility preference. Furthermore, educational status and region were community factor associated significantly with intention of fertility. Hence, expansion of mobile networking and family planning messages through mobile. Furthermore, religious teaching should be enhanced to control family size among followers. Finally, the Ethiopian government should also work strongly to improve community education.

## Background

Fertility is one of the three pillars of events which determine the overall population size, and structure of one country [1]. In most sub-Saharan African countries, fertility rate reduction was not achieved as expected with the exception of some communities ,and metropolitan areas [2, 3].In Ethiopia, the total fertility rate was declined from 5.9 children per woman to 5.4 children per woman from 2000 to 2005 [4]. The population growth rate of one country is closely related with its sustainable growth, and development. Therefore, Ethiopia could grow economically only if its population growth rate is managed appropriately [5, 6]. Therefore, fertility regulation should be the primary concern of public health authorities and local governors in Ethiopia [5].

In Ethiopia, rapid population growth has challenged its growth and development. For example, poverty, war, and famine have resulted into many adverse effects: Inadequate education, poor health service, weak infrastructure, and low agriculture products [7, 8]. Nearly half of the Ethiopian population is economically dependent on population of productive age group on both sexes. This population group is highly influenced by additional collateral problems such as: Unemployment, underemployment, and physical disability. Hence, increased age dependency ratio has persisted in Ethiopia for a long time period [7, 9].

In rural Ethiopia, the total fertility rate among married women was declined from 6 children in 2000 to 5.2 children in 2016. Furthermore , it was declined from 3 children in 2000 in rural areas to 2.3 children in 2016 in urban areas [10]. Although fertility rate has been declined remarkably, it was not equally distributed equally among regions [11]. Fertility desire is the first step which determine fertility behavior and level [12]. Based on the report of previous studies, socio-demographic and reproductive factors were associated with fertility preference. For example, marriage status was one of the predictor of fertility outcome [13]. Moreover, studies done in Sub-Saharan African countries have indicated that being unmarried was associated with decline of fertility [14, 15]. The status of fertility is the result of well-organized biological and behavioral factors. These factors are moderated indirectly on culture, socio-economic, and living standards of the given society [16].

The national population policy was drafted in Ethiopia in 1993 which targets the control of population [17–19]. According to HSDP (Health Sector Development Plan), Ethiopian government has targeted to increase contraceptive prevalence rate to more than 55% and reduce unmet need of contraceptive to 10% by the end of 2020 [20]. A lot of mixed factors at varies levels were associated with fertility intention. Lack of education, poverty, rural residency, and low husband educational status were associated with declining of fertility intention. Furthermore, the reproductive health charactestics like: Early mirage, history of child death, and negative attitude of husband on contraceptive utilization were associated with high fertility [21]. On the contrary, increased age, knowledge and utilization of modern contraceptives, media exposure for contraceptive ,more number of living children were associated with low intention of fertility [22]. Several community level factors were associated with intention of fertility. For example, access to family planning methods, female education, religion, residence, and media exposure [23, 24] were factors associated with improving fertility intention.

On the other hand, average community wealth index has significant positive effects on the use of modern contraceptives [25]. By implication, women who are in a community with more chance of family planning service have less chance of intention for fertility. Behavioral intentions are the final common pathway through which fertility motivations, attitudes, beliefs, and desires affect actual fertility [26]. Although majorities of previous studies have focused on family planning service utilization among married women who did not intended to be fertile [27–32]. Contrary to this fact, information is scarce about fertility intention at various level in Ethiopia. The result from these study provided wide support in formulation of better population and demographic policy and program as a nation by highlighting factors which initiate fertility desire. It can inform the local governors and health providers to strengthen the collateral health services. This study aimed to assess prevalence and factors associated with fertility intention among married women in Ethiopia in 2016.

## Method

### Study design, study area, and period

This study was a secondary data analysis of a population based cross sectional study of 2016 Ethiopian demographic health survey. The study was conducted in Ethiopia. Ethiopia lies between 30 and 150 North latitude and 330 and 480 East longitudes. It is divided into nine regional states, and two city administrations. These regional states and administrations are further sub-divided into 75 zones, 551 districts and 10,000 Kebeles (the smallest administrative units in Ethiopia) [33]. In Ethiopia, health sector development Plan-I have introduced having a four-tier health system for health service delivery. It consists of different level of health institutions from lowest to highest: One health center and five satellite health posts, district hospital, zonal hospital, and specialized hospitals.

### Data source, study population and sampling technique

**Fig. 1:**
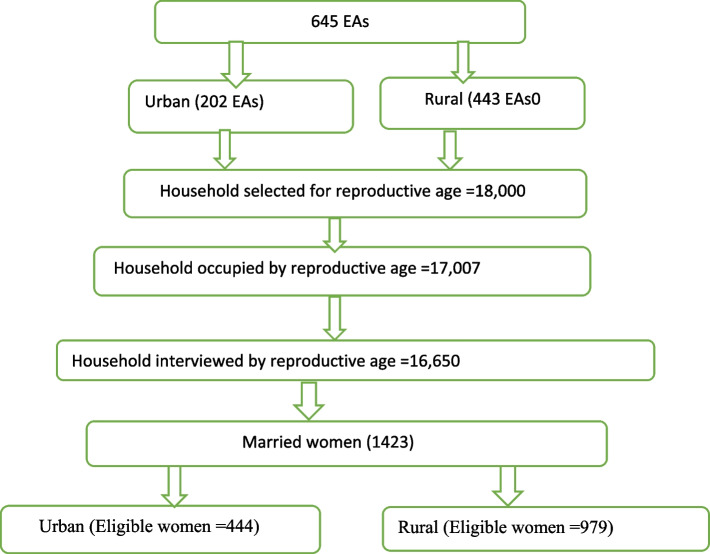
Schematic presentation of sampling strategy of intention of modern contraceptive among married women in Ethiopia (EDHS 2016)

This study was based on the Ethiopian demographic and health survey (EDHS) 2016 data which was a nationally representative sample conducted from January 18, 2016, to June 27, 2016. To select enumeration areas for EDHS 2016, a total of 84,915 Enumeration areas (EAs) from an Ethiopian Population and Housing Census (PHC) conducted in 2007 were used as a sampling frame. Regarding the sampling technique, the survey used a two-stage stratified cluster sampling technique selected in two stages**.** Regarding the sampling technique, the survey used a two-stage stratified cluster sampling technique selected in two stages. In the first stage a total of 645 EAs (443 in rural areas) and in the second stage an average of 28 households per each cluster were selected. Any further information about the data/survey exists in the 2016 EDHS report [34]. For this study, we have used the individual data set and the study population was all women (aged 15 to 49 years) (See Fig. 1).

### Study variables

Fertility intention is the binary outcome variable in this study. In the DHS data set fertility intention was described as nominal variable with five options: have another intention for fertility, undecided, no more intention, sterilized (respondent or partner), and declared infecund. The author has categorized these options into two categories. Hence, the outcome variable was binary outcome. It was labeled as ‘0’ and ‘1’ for non-intention and intention of fertility respectively. Women who reported as “undecided “sterilized”, “infecund” and “no more” were categorized as having no intention of fertility in the analysis of this study. Both individual and community level variables were included in this study. The individual level variables included in this study were grouped under the following categories: Socio-demographic, reproductive, and source of information variables.

The socio-demographic variables were: Age, educational status, wealth index, region, working status, religion, ethnicity, and residence. In addition, reproductive variables included in this study were: Age of 1^st^ cohabitation, intention to use modern contraceptive, modern use contraceptives, knowledge of modern contraceptive methods, number of union and ever terminated pregnancy. Furthermore, variables classified under the source of information were own mobile, heard information by text message, reading newspaper, watching television, listening radio and use of internet. We have utilized principal component analysis to construct wealth index. Different type of house hold assets are variables used in principal component analysis. Thus include size of production land, livestock and house building material. Hence, wealth quantile is divided into five levels: Poorest, poorer, medium, richer and richest [35]. Wealth index was created in three consecutive steps. First, the subset of urban and rural households’ indicators was built separately. Second, scores were created separately for each household both in urban and rural. Finally, a national wide wealth index was created by combination of both urban and rural [36].

The community level variables included in this study were: Educational status, wealth index, access for family planning, media access and place of residence. The community level variables were created by aggregating the individual woman value of respective variable at cluster level. Then, the aggregated values were categorized as binary outcome variable. Those value below and above the mean level was conducted yielding the lowest and highest value for the above community level variables. Hence, clusters were categorized as “0” and “1” for lower and high value of the respective variables. For example, those clusters who scored below the mean value of aggregated value for education were considered as “low education” and labeled as “0”. On the contrary, clusters which scored above the mean were considered as “high education” and labeled as “1”.

### Analysis and model selection

Data were entered, coded and cleaned using SPSS version 22 from the source. Then, data was re-categorized, coded and labeled variables to make them comparable across countries. Finally, data was exported into STATA 14 and analyzed. However, important variables were selected through comprehensive literature reviews [37–39]. The data for this study came from complex survey. Ideally, both bias and variance should be kept minimum in complex survey. Therefore, sampling weight was applied to minimize bias and compensate for unequal probability of selection among geographic strata. Weight variable was created by dividing variable V005 by 1,000,000 in this study. The author has checked the extent of outliers, the different statistical assumptions, and applied the appropriate correction mechanisms prior to analysis. In this study, women characteristics were considered as the lower (first) level factors. On the other hand, cluster level (secondary) level factors were regarded as the highest level variables in this study.

The following equation elaborates multi-level analysis of fertility for desire

Logit (Pij)=b0+b1Iij+b2Cj+UJ+єij:

Where, i and j are the level 1 (individual) and level 2 (community) units, respectively. Furthermore, I, and C refer individual, and community level variables, respectively. Pij is the probability of intention of fertility for the i^th^ woman in the j^th^ community. The ‘b’s indicate the coefficients. Where ‘b0’ is the intercept. The intercept ‘b0’ is the effect on the probability of intention of fertility in the absence of independent variables; and Uj showed the random effect (effect of the community on intention of fertility for the jth community) and єij showed random errors at the individual woman level or unmeasured factors that may influence fertility desire.

By assuming each community had different intercept (b0), and fixed coefficient (b), the clustered data nature and the within and between community variations were taken into account. Then, the analysis was performed using four models. Therefore, model 0 (empty model or null model/ without explanatory variable/), model 1 (Only individual level factors), model 2 (only community factors) and model 3 (both individual and community level factors).

The measures of variation (random-effects) were reported using ICC, X^2^ test and proportional change in variance (PCV) to measure the variation between clusters. The ICC shows the variation in intention of fertility married women due to community characteristics. The ICC was calculated as follows: [ICC= δ2 δ2þπ32], where δ2 is the estimated variance of clusters.

The higher the ICC, the more relevant was the community characteristics for understanding individual variation in intention of fertility for married women. The data showed that there was correlation at clustered level that obligate to use multilevel analysis model. Furthermore, VPC measures the total variation attributed by individual-level factors and community-level factors in the multilevel model. The variance coefficients were assessed as shown: VPC = σ2 uo/ (σ2 uo+ π2/3), where, π2/3 denotes the variance between mother from the same cluster (individual-level) and σ2u0 is the variance between cluster (community-level variance). It gives how much of the variance is explained at the community-level [40].

In this study, first, bivariate multilevel logistic regression was fitted in model 1 and 2. Then, variables with p-value less than 0.25 were selected to build model 3. The measures of association (fixed-effects) estimate the associations between the likelihood of married women to have intention of fertility and various explanatory variables were expressed as Adjusted Odds Ratio (AOR) with their 95% confidence level. In model 3. Variables whose p-value < 0.05 were reported as the final variables which predicts the fertility desire. Multi-collinearity and interaction effect checks were also done by measuring Variance Inflation Factors (VIF) was more than 2 (acceptable), labelling of outliers and running cross products. Multi-collinearity and interaction effects were not observed among the variables included in the models.

### Model fitness

The log likelihood test was used to estimate the goodness of fit of the adjusted final model in comparison to the preceding models (individual and community level model adjustments). Based on Akaike information criteria (AIC) result used to select the final fitted model. Likelihood ratio test vs. negative binomial model was significant at Chi-square < 0.001. For bivariate mixed effect negative binomial regression P-value < 0.25 at 95%CI was taken as a significant and P-value <5% at 95%CI was takes as significant value for multivariable mixed effect negative binomial regression. Moreover, chi-square test (X^2^) was performed to observe any association between independent variables and outcome variable. Descriptive statistics was conducted by the use of texts and tables. Multi-level analysis can address the two main problems that can occur either due to aggregation or disaggregation of data. First, if data are aggregated we loss data and power. Second, if data are disaggregated and are not independent of one another, it can lead to false positive significant effect when in fact not exist [41].

## Results

Table 1 presents the socio-demographic characteristics of the study participants in this study. All women, who were selected for this study, have given fully response making the non-response rate as 100%. Accordingly, the mean age of the study participants was 18+ 3.8 years. More than half (56.6%) of the study subjects were not formally educated. Furthermore, majorities of the study participants (71.6%) had their own work. Most of women (68.8%) of the study participants were rural dwellers. Furthermore, women in Amhara, Oromia and Tigray region were 474(33.3%), 446(31.4%), and 189(13.3%) respectively.

**Table 1:** Socio-demographic characteristics of married reproductive age group women in Ethiopia 2016 EDHS, 2019(1423)

Variables	Category	Unweight n(%)	Weighted n(%)
Age of women	15-19 years	664 (6.8)	151 (10.6)
	20-34 years	1783 (18.1)	605 (42.3)
	35-49 years	5879 (59.8)	667 (46.9)
Educational status	No education	3287 (33.5)	805 (56.6)
	Primary education	2700 (27.5)	456 (32)
	Secondary education	880 (9)	110 (7.8)
	Higher education	551 (5.6)	52 (3.6)
Working status	Work	5345 (54.4)	1019 (71.6)
	No work	450 (45.6)	404 (28.4)
Place of residence	Urban	2491 (25.4)	444 (31.2)
	Rural	7333 (74.6)	979 (68.8)
Region	Tigray	957 (9.7)	189 (13.3)
	Afar	866 (8.8)	13 (0.9)
	Amhara	1128 (11.5)	474 (33.3)
	Oromia	1317 (13.4)	446 (31.4)
	Somali	978 (10)	34 (2.4)
	Benishangul	806 (8.2)	11 (0.8_
	SNNPRs	1217 (12.4)	137 (9.6)
	Harari	576 (5.9)	4 (0.3)
	Gambela	712 (7.2)	5 (0.3)
	Addis Ababa	677( 6.9)	97 (6.8)
	Dire Dewa	590 (6)	12 (0.9)
Religion	Orthodox	3535 (36)	831 (58.4)
	Muslim	4318 (44)	367 (25.8)
	Others^a^	1973 (20)	226 (15.9)
Wealth index	Poorest	2998 (30.5)	267 (18.7)
	Poorer	1776 (18.1)	210 (14.8)
	Middle	1753 (17.8)	267 (18.8)
	Richer	1614 (16.4)	243 (17.1)
	Richest	1683 (17.1)	436 (30.7)
Ethnicity	Amhara	2108 (21.5)	614 (43.2)
	Oromo	2328 (23.7)	387 (27.2)
	Tigre	1054 (10.7)	197 (13.9)
	Others^b^	4334 (44.1)	225 (15.8)
Region	Tigray	282 (17.8)	189 (13.3)
	Afar	96 (6.1)	13 (0.9)
	Amhara	219 (13.9)	474 (33.3)
	Oromia	145 (9.2)	446 (34.1)
	Somali	111 (7)	34 (2.4)
	Benishangul	80 (5.1)	11 (0.8)
	SNNPR	76 (4.8)	137 (9.6)
	Gambela	117 (7.4)	5 (0.3)
	Harari	103 (6.5)	4 (0.3)
	Dire-Dawa	190 (12)	97 (6.8)
	Addis Ababa	162 (10.2)	12 (0.9)

**Key:** ^a^Protestant, catholic, traditional belief, ^b^Somali, Sidama, wolayta, Gedio, Hadiya, Afar, Gumuz, keficho, Berta, Guarage, Siltie, Yem, Burji, Aniwak, Goffa, Argoba, Mejenger, Agew, Alaba, Derashe, Dezi and Wolayta, SNNPR- Southern Nation Nationalities and Peoples Representative

Majority of the study subjects (58.4%) were followers of orthodox Christianity religion. On the contrary, only fifteen point nine (15.9%) study participants were followers of other religions: (Protestants, catholic and traditional belief). Additionally, nearly one-third of the study subjects were grouped under richest wealth index (See Table 1).

According to Table 2 report, majorities of the study subjects (84.5%) started living together with their partner by the age of less than 18 years. Moreover, only 512(36%) of the study participants had future fertility need. Furthermore, only 208(14.6%) of the study subjects were utilizing modern contraceptive method. But, almost all 1403(98.5%) of the study participants had knowledge of modern contraceptive methods. In Ethiopia, significant number of study subjects (30.7%) still had more than one partners. Only 131(9.2%) study subjects had abortion in this study. Furthermore, Table 2 also indicate that nearly one-third (31.7%) of the study subjects were mobile users. But, few 24(1.7%) study subjects were received family planning message through mobile phones. One from four study subjects listens radio. Only 43(3%) of the study subjects use internet service. Almost one third 428(30.1%) of the study subjects were watching television (See Table 2).

**Table 2:** Reproductive health and source of data variables of married reproductive age group women in Ethiopia 2016 EDHS, 2019

Variables	Category	Unweight n(%)	Weighted n(%)
Age of 1^st^ cohabitation	Less than 18 years	5938 (60.44)	1203 (84.5)
	More than 18 years	3886 (39.56)	220 (15.5)
Fertility preference	Yes	6927 (70.5)	512 (36)
	No	2897 (29.5)	911 (64)
Current user of modern contraceptive	Yes	2900 (29.5)	208 (14.6)
	No	6924 (70.5)	1215 (85.4)
Knowledge of modern contraceptive	Yes	9395 (95.6)	1403 (98.5)
	No	429 (4.4)	20 (1.4)
Number of union	One	8269 (84.2)	986 (69.3)
	More than one	1559 (15.8)	437 (30.7)
Ever terminated pregnancy	Yes	8778 (89.4)	1292 (90.8)
	No	1046 (10.6)	131 (9.2)
Own mobile	Yes	6885 (70.1)	451 (31.7)
	No	2939 (29.9)	973 (68.3)
Heard information by text message	Yes	9636 (98.1)	24 (1.7)
	No	188 (1.9)	1399 (98.3)
Reading news paper	No	8786 (89.4)	1267 (89)
	Yes	1038 (10.6)	156 (10.9)
Watching television	No	2728 (72.2)	995 (69.9)
	Yes	1768 (18)	428 (30.1)
Listening radio	No	6924 (70.5)	1049 (73.7)
	Yes	2900 (29.5)	374 (26.3)
Use of internet	Yes	426 (4.3)	43 (3)
	No	9398 (95.7)	1380 (97)

### Fixed-effect model

Model 4 of Table 3 describes the adjusted individual, and community level factors associated with fertility desire. Crudely associated variables, which were identified by bivariate logistic regression model, were age of the study participants, region, intention to use modern contraceptive, frequency of reading newspaper, watching Television, listening radio, internet use, and number of children. After adjusting the cofounders, the individual woman variables were age, religion, own mobile, lack of intention for modern contraceptive utilization. Moreover, community educational status and region were the community variables which had a statistically significant association with fertility intention in the multivariate logistic regression analysis model.

**Table 3:** Multi-level mixed effect logistic regression on intention of contraceptive utilization among married women in Ethiopia EDHS 2016 dataset, 2019

Individual level variables	label	Model 0 ICC=28.8%	Model I COR(95%CI)	Model II COR(95%CI)	Model III AOR(95%CI)
Age (years)	15-19		Ref.		Ref.
	20-34		1.9 (1.1, 3.3)*		1.9 ( 1.1, 3.3)**
	35-49		28.4 (15.3, 52.8)*		23.6 (12.2 45.4)**
Religion	Orthodox		Ref.		Ref.
	Muslim		3.1 (2.3, 4.3)*		5.4 (3.6, 7.9)**
	Others*		2.5 (1.70, 3.8)*		1.8 (1.2, 3.0)**
Intention to use modern contraceptive	No		Ref.		Ref.
	Yes		7.3 (5.4, 9.8)*		3.1( 2.2, 4.3)**
Reading newspaper	No		Ref.		Ref.
	Yes		0.43 (0.30, 0.6)*		0.70 (0 .43,1.1)
Listening radio	No		Ref.		Ref.
	Yes		0.56 (0.42, 0.74)*		0.78 (0.54, 1.1)
Watching TV	No		Ref.		Ref.
	Yes		0.81 (0.69, 0.95)*		0.93 (0 .64, 1.3)
Own mobile	No		Ref.		Ref.
	Yes		0.55 (0 .41, 0.73)*		0.9 (0 .68, 1.4)
Number of union	One		Ref.		Ref.
	More than one		1.3 (1.02, 1.8)*		0.5 (0.41, 0.8)**
Number of children	No		Ref.		Ref.
	Yes		1.4 (1.2, 2.15)*		1.0 (0.5, 1.7)
Internet use	No		Ref.		Ref.
	Yes		0.20 (0.11, 0.38)*		0.56 (0.28, 1.16)
**Community variables**					
Education	High			Ref.	Ref.
	Low			1.61( 1.26, 2.0)*	1.67 (1.26,2.20)**
Region	Tigray			Ref.	Ref.
	Afar			0.61 (0,35,1.05)	0.66 (0.38, 1.15)
	Amhara			0.93 (0.6,1.43)	1.07 (0.69, 1.65)
	Oromia			0.34 (0.20,0.57)*	0.37 (0.22,0 .61)**
	Somali			0.82 (0.49, 1.38)	0.81 (0.56, 1.57)
	Benishangul			0.70 (0.39 ,1.27)	0.74 (0.41, 1.34)
	SNNPR			0.26 (0.13,0.52)*	0.29 (0.15, 0.58)**
	Gambela			0.45 (0.26, 0 .78)*	0.41 (0.24, 0 .72)**
	Harari			0.31 (0.17, 0.57)*	0.27 (0.15, 0.50)**
	Dire-Dawa			1.1 (0.73, 1.78)	0.91 (0.58, 1.43)
	Addis Ababa			0.79 (0.49, 1.29)	0.75 (0.46, 1.23)

**Key:** Others*-catholic, Protestant and traditional beliefs

Specifically, woman age from 20-34 years were 2.5 times more likely than age of 15-19 years (AOR=2.5,95%CI, 1.53, 4.0). Women age from 35-49 were 23.6 times more likely than women of age of 15-19 years (AOR= 9,95%CI, 12.2,45.4). The odds of fertility need among Muslim women were 5.4 times more than women of Orthodox Christians (AOR=5.4,95%CI,3.6,7.9). The chance of fertility preference among study participants who follow others religions were 1.8 times more than orthodox Christian participants (AOR= 1.8,95%CI,1.2, 3.0). Study subjects who own mobile were 40% less likely than their counterparts (AOR= 0.6,95%CI,0.4, 0.87). Study subjects who did not intend to use modern contraceptive were 3.5 times more likely to intend to be fertile methods than their counterparts (AOR=3.5,95%CI,2.53, 4.84). Regarding community variables, educational status, and regions were factors associated with fertility desire. Therefore, married women who lived in communities with low education status were 1.7 times more likely desire fertility than their counterparts (AOR=1.7, 95%CI,1.2, 2.2). Region is another key variable correlated with desire of fertility: Women in Oromia region was 63% less likely to desire fertility than Tigray region (AOR=0.37,95%CI, 0.22,0 .61), women in South nation nationality and peoples of Ethiopia region were 81% less likely to intend fertility than Tigray region (AOR=0.29 ,95%CI, 0.15, 0.58). Additionally, women in Gambela were 59% less likely to desire fertility than women in Tigray region (AOR=0.41,95%CI, 0.24, 0.72). Finally, women in Harari were 63% less likely than women in Tigray region in desiring more fertility (AOR=0.27, 95%CI, 0.15, 0.50) (See Table 3).

## Discussion

In recent years, the population growth rate is increasing alarmingly in Ethiopia. Unless it is managed appropriately, the high population growth can be amplified by pre-existing natural and human made disasters. As a result, it could result in national crisis by creating huge burden on scarce resources. Intention to perform certain behavior is assumed to be the first step for performance of that behavior [12]. Furthermore, the intention to have more children is not only associated with individual level factors but also it is highly influenced by the community factors in which women born, grow and lives. Although a numbers of local studies were done on fertility and its associated factors in Ethiopia, national information is scarce which highlight multi-level factors of fertility intention. These limitations are effectively addressed in this study.

Prevalence of intention of fertility was found to be 63.5% (95%CI :62.2%-64.8%) among married women of age 15-49 years in Ethiopia. Moreover, the explanatory variables included in this study were behavioral and biological factors. Therefore, the cultural and behavioral factors are still being consistent in Ethiopia to yield high fertility rate and low contraceptive utilization intention in Ethiopia [42]. The heterogeneity of Ethiopian population in religion, culture and socio-economic factors might bring high fertility need in Ethiopia in this study.

Regarding specific individual level factors associated with fertility preference in this study, Older women were more likely prefer more children than their counterparts. The current finding is inconsistent with the previous study in Hawassa, Ethiopia [43]. The possible inconsistency might be due to variation in the scope of the study. The finding of this study was aggregated finding of the country. Women who had intention to use modern contraceptives had less chance of getting pregnancy than their counterparts. This finding is similar with previous study conducted in Ethiopia [43, 44]. The possible explanation might be that women who intend to use modern contraceptive do not have desire of fertility. Furthermore, socio-economic and cultural charactestics of women were similar with the study subjects in the current study. According to previous study report, utilization of contraceptives were the main predictors of future fertility desire [45].

The individual factors associated with fertility intention were found to be wealth index, religion, number of partners, and marital status. Specifically, the current study indicates that women in high wealth index had more odds of having fertility intention than their counterparts. This finding is in line with findings of previous studies in in Ethiopia [46, 47]. The possible explanation might be that the rich women had better chance of access and utilization of modern contraceptives. A study conducted in china indicated government expenditure increases on fertility intention [48]. Previous study finding indicated that women with high income is associated with more contraceptive utilization [49].

Furthermore, religion is one of the significant factor in this study. For example, Muslim religion and traditional beliefs were associated with high fertility than orthodox Christianity. This finding is in line with previous studies [50, 51]. The possible rational for this similarities might be associated with fertility differentials across religious groups are usually explained with the content of religious teachings that affect demographic behaviors. Even though not all religions have direct rules and teachings on reproductive behaviors, such as contraception and abortion, general teachings on values and attitudes toward family, gender roles, and childbearing can affect fertility behaviors in indirect ways. The characteristics approach attributes fertility differentials by religion to difference in socio-economic [52]. Based on the report of this study, women who have multiple sexual partners were more likely to desire fertility than their counterparts [53]. The possible rational could be explained by the fact that women with multiple partners could start their relationship in earlier than their counterparts [54]. This might be due to their high fertility need. This study indicate that higher age is also associated with more fertility preference. This finding is consistent with the previous finding in Ethiopia [55]. The possible explanation might be explained that as the age of women increases the fertility capacity reduced [56]. Furthermore, women become economically secured and fear of being menopause with few children elevate the desire of fertility.

Education and region were community factors associated with fertility intention. Women who live in communities with low education status were more likely to intend future fertility than their counterparts. This might due to the fact that women who live in communities with low level of literacy did not utilize contraception among women communities [57, 58]. Women in other regions were more likely to desire fertility than women in Tigray region. The possible explanation might be due to low level of knowledge and attitude of modern contraceptive rate among women in these regions. Previous evidence proved that women who live in communities with little awareness about modern contraception did not use modern contraception as a result they tend to desire more children [59].

### Strengths and limitations of the study

The finding of this study is generalizable due to large sample size, national wide study and using standard questionnaire. However, this study has a number of limitations. For example, it was cross-sectional study. As such, the study was unable to conclusively determine the temporal relationship between the explanatory variables and outcome variable. Furthermore, some cultural and health institution factors were not explored in this study.

### Conclusions and recommendation

To conclude, fertility intention was high among married women in Ethiopia. The policy makers, program designers and stakeholders should increase effort to enhance good attitude towards limiting number of children, strong awareness creation against traditional custom in the community and avoiding barriers for more fertility intention. Hence, expansion of mobile networking and family planning messages through mobile. Furthermore, religious teaching for control of family size should be enhanced. The government should also work strong work to improve community wealth. Finally, future researchers might further explore the reason of having more intention for fertility.

### Implication of the study

This study can have many public health implications. First, this study highlights the benefit of developing and implementing policy to improve knowledge of impact of high fertility need.

Second, improving the socio-economic status of the population is mandatory among women to minimize habit of high fertility need. As a result, the local government should focus for improving the standard of living of married women.

## Data Availability

Permission to access database was officially obtained. The database was available at a official website of DHS which is at https://dhsprogram.com.

## Abbreviation

AOR: Adjusted Odds Ratio
COR: Crude Odds Ratio
ICC: Intra-Cluster Correlation
SNNPR: Southern Nation Nationalities and Peoples Regional State
EDHS: Ethiopian Demographic Health Survey
EAs: Enumeration Areas
CSA: Central Statistics Agency
FMOH: Federal Ministry of Health
EPHI: Ethiopian Public Health Institute
AIC: Akaike Information Criteria
VPC: Variance Partition Coefficient
